# Immune Markers Linking Chronic Periodontitis with Gestational Complications: A Systematic Review

**DOI:** 10.12669/pjms.41.11.12722

**Published:** 2025-11

**Authors:** Ayesha Sadiqa, Zahra Sakina, Faiza Fatima Chishti

**Affiliations:** 1 Ayesha Sadiqa Department of Physiology, Institute of Dentistry at CMH Lahore Medical College and Institute of Dentistry, Lahore, National University of Medical Sciences (NUMS), Rawalpindi, Punjab, Pakistan; 2 Zahra Sakina Department of Periodontology, Institute of Dentistry, Institute of Dentistry at CMH Lahore Medical College and Institute of Dentistry, Lahore, National University of Medical Sciences (NUMS), Rawalpindi, Punjab, Pakistan; 3 Faiza Fatima Chishti Department of Community & Preventive Dentistry, Institute of Dentistry, Institute of Dentistry at CMH Lahore Medical College and Institute of Dentistry, Lahore, National University of Medical Sciences (NUMS), Rawalpindi, Punjab, Pakistan

**Keywords:** Chronic periodontitis, Gestational diabetes mellitus, Immunological Markers, Low birth weight, Pregnancy outcomes, Preeclampsia, Premature birth

## Abstract

**Objective:**

This systematic review aimed to determine how mediators establish a causal association between chronic periodontitis and adverse pregnancy outcomes (APOs).

**Methodology:**

The PRISMA 2020 guidelines were followed. Studies were selected using the PECO model to assess the inflammatory mediators linking chronic periodontitis and APOs. Six databases (PubMed, Scopus, Web of Science, Embase, Cochrane Library (CENTRAL) and Google Scholar) were searched from May 15-20, 2025. Inclusion criteria targeted English-language studies (2000-2025) reporting inflammatory markers in periodontitis-affected pregnancies. Case reports, editorials, conference abstracts, grey literature, in vitro studies and studies involving subjects other than humans, rats and baboons were excluded. Two independent reviewers screened and extracted data. The risk of bias was assessed using ROBINS-I, NOS or ROBIS tools. A qualitative synthesis of findings was performed.

**Results:**

Periodontitis is strongly linked to APOs, including gestational diabetes mellitus (GDM), preterm deliveries, preeclampsia and low birth weight (LBW). Inflammatory mediators, such as TNF-α, IL-1β, IL-6, IL-8, CRP, PGE2, MCP-1 and MMPs, are elevated, disrupting placental function.

**Conclusion:**

Periodontitis is strongly associated with APOs through direct microbial invasion and indirectly through systemic inflammatory mediators, which disrupt placental function and the fetus’s development. These findings highlight that a healthy periodontium is crucial in preventing complications like GDM, preeclampsia, preterm deliveries and LBW.

## INTRODUCTION

Chronic periodontitis, a multifaceted inflammatory disease, destroys tissues supporting the teeth, collectively known as the periodontium.^1^ Periodontal pathogens, primarily gram-negative bacteria, destroy periodontal tissue in a susceptible host, resulting in clinical attachment loss (increasing the sulcus depth), irreversible bone loss (osseous resorption) and, in adverse cases, tooth loss (exodontia).^2^ As periodontitis progresses, chronic inflammation translocates periodontal bacteria and their by-products into the bloodstream, resulting in systemic ill effects.^2^,^3^ Anaerobic bacteria, along with their end products, are placed in periodontal pockets in chronic periodontitis and this bacterial reservoir can invade other body tissues directly or indirectly. That’s why periodontal infection has been related to systemic abnormalities in a maternal body with high oxidative stress, including preterm birth.^4^,^5^ Due to its systemic effects, numerous studies have linked periodontitis to chronic inflammatory conditions, namely diabetes, cardiovascular diseases and rheumatoid arthritis.^6^,^7^

The same connection was also explored through animal studies, where periodontitis in pregnant baboons has been linked to APOs, including preterm deliveries, LBW, spontaneous abortion, stillbirth and fetal demise.^8^ Similarly, translocation of *P. Gingivalis* to pregnant rodents’ placenta has been shown to elevate IL-6, IL-17, IL-1β and TNF-α, resulting in degenerative changes and preterm deliveries.^9^

Two potential pathways (direct and indirect) have been proposed to explain the association between APOs and periodontitis.^10^ Periodontal pockets store bacteria, which can enter the bloodstream, travel to the placenta,^10^,^11^ and trigger metastatic infections. The direct pathway involves the translocation of periodontal bacteria from periodontal sites to the fetoplacental unit (FPU). Here, periodontal bacteria, including *Porphyromonas gingivalis* and *Campylobacter rectus*, have been found in the amniotic fluid of expectant mothers who have periodontal disease.^7^,^10^,^12^ The indirect pathway is mediated via inflammatory mediators, namely interleukins such as IL-1, IL-6, IL-8, TNF-α and prostaglandins like PGE2 from periodontal sites, triggering a host immune response. These mediators may initiate a systemic response by upregulating C-reactive protein (CRP) production that will ultimately cause APOs.^8^,^13^

Literature has identified several periodontal pathogens within FPU, such as *Porphyromonas gingivalis, A. actinomycetemcomitans, Parvimonas micra, Filifactor alocis, Prevotella intermedia, C. rectus, Treponema denticola and F. nucleatum*.^10^,^12^,^14^ Movement of *P. Gingivalis* to the placenta in pregnant rodents has been shown to elevate TNF-α, IL-1β, IL-6 and IL-17 levels, contributing to intrauterine inflammation.^15^ Additionally, periodontal inflammation causes elevation of pro-inflammatory cytokines like PGE2, TNF-α, IL-1 and IL-6, that enter the systemic circulation.^6^,^16^ Although evidence suggests a strong association, the exact mechanism remains unclear due to multiple confounding factors such as age, race and socio-economic background.^14^,^17^

Micro-ulcerations within periodontal pockets significantly compromise the integrity and selective permeability of the junctional epithelium, thereby facilitating the movement of periodontal pathogens and their derivatives into the blood circulation and enabling their dissemination throughout the body.^8^,^18^ Numerous epidemiological studies expressed an association of periodontitis with preterm birth, gestational diabetes and preeclampsia.^19^,^20^ Thus, the objective of this systematic review was to find out whether chronic periodontitis has been associated with adverse pregnancy outcomes (APOs), what mediators mediate this link and what mechanisms are behind it.

## METHODOLOGY

This review was carried out in accordance with the Preferred Reporting Items of Systematic Review and Meta-Analysis (PRISMA) 2020 guidelines for systematic reviews.^21^ It was a prognostic systematic review assessing the adverse gestational outcomes in individuals with periodontal disease and registering it was not financially feasible.

### PECO Question:

The following research question was answered as per the PECO model (Table-I):^22^ “In adult pregnant females (humans, baboons or rats), does the presence of periodontitis and associated systemic inflammation, compared to healthy periodontal status, increase the risk of adverse pregnancy outcomes and alter levels of inflammatory mediators such as cytokines and prostaglandins?”

**Table-I:** PECO question for a systematic review of inflammatory mediators that connect periodontitis to adverse pregnancy outcomes.

Population (P)	Pregnant adult females (humans, baboons or rats) of any age
Exposure (E)	Chronic Periodontitis in gestation and its associated adverse pregnancy outcomes (APOs)
Comparison (C)	Pregnant women without periodontitis (healthy periodontal status)
Outcome (O)	Mediators associated with APOs confirmed via elevated clinical body fluid (serum, blood, amniotic fluid, gingival crevicular fluid) profile or count of such interleukins, chemokines, cytokines or adhesion molecules

### Eligibility Criteria:

### Inclusion criteria:

- Primary human or animal studies (observational or interventional), reviews and meta-analyses examining the link between periodontitis and gestational outcomes in adult females.
- Studies reporting levels or roles of inflammatory mediators (e.g., cytokines, prostaglandins) in pregnant subjects with both periodontitis and APOs.
- Studies published from the year 2000 to the year 2025.
- Only peer-reviewed articles in English.

### Exclusion criteria:

- Case reports, editorials, conference abstracts and in vitro studies.
- Studies involving subjects other than humans, rats and baboons.
- Studies not reporting specific inflammatory mediators or not focusing on pregnancy outcomes.
- Non-English publications and grey literature.

### Information Sources & Search Strategy:

PubMed, Scopus, Web of Science, Embase, the Cochrane Library (CENTRAL) and Google Scholar were explored from 15th to 20th May 2025 and 343 records were retrieved. Search strings were tailored to each database but covered the same concepts. Additional sources included hand-searching reference lists of relevant articles. Searches included only English-language records were considered. PRISMA-S recommendations were followed to report search strategies, as shown in Table-II.

**Table-II:** Search strategies and number of studies from various databases.

Database	Search strategies	Number of studies
PubMed	Search terms included (“periodontitis” OR “periodontal disease”) AND (pregnancy OR gestation OR “pregnancy complications” OR preeclampsia OR “preterm birth”) AND (“inflammatory mediator*” OR cytokine* OR chemokine* OR TNF OR “interleukin-6”)	90
Scopus	Similar keywords as PubMed applied to title/abstract fields	75
Web of Science	Topic search with (periodontitis OR periodontal) AND (pregnancy OR gestational) AND (inflammation OR cytokine OR chemokine)	60
Embase	Search terms (periodontitis OR ‘periodontal disease’) AND (pregnancy OR gestation OR “low birth weight” OR preeclampsia) AND (inflammation OR cytokine OR chemokine)	83
Cochrane Library	CENTRAL search using (periodontal AND (pregnancy OR preeclampsia) AND inflammation)	2
Google Scholar	General search on “periodontitis pregnancy inflammation cytokines preterm,” screening the first 100 results	33

### Selection Process:

**Fig.1:**
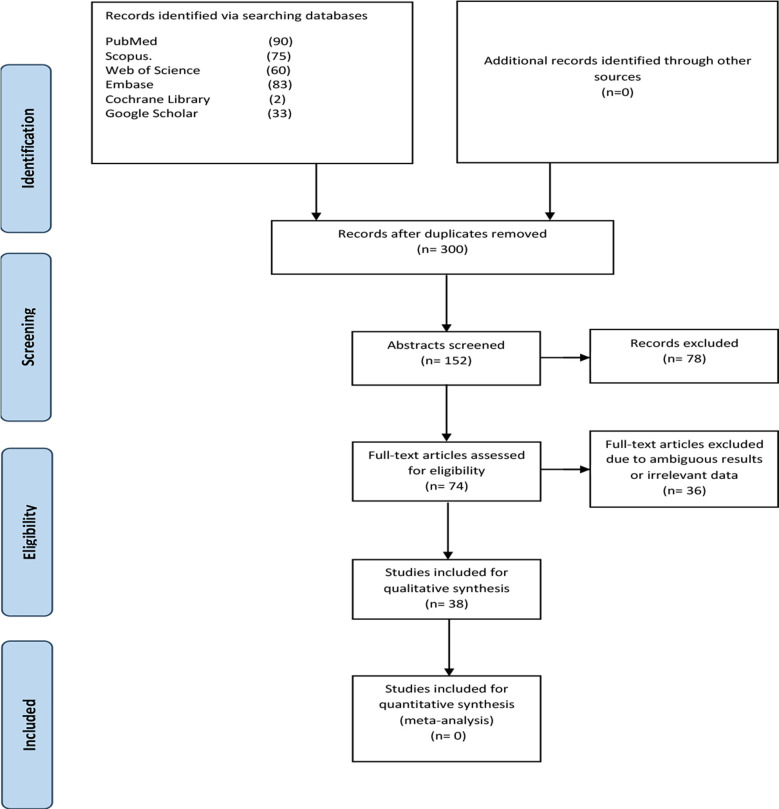
Steps of article selection based on PRISMA flowchart.

Two reviewers (Ayesha Sadiqa and Zahra Sakina) independently screened titles and abstracts, resolving disagreements by discussion or third-party involvement (Faiza Fatima Chishti). After the full article screening and risk-of-bias assessment (Fig.1), 38 studies were included for analysis.

**Fig.2:**
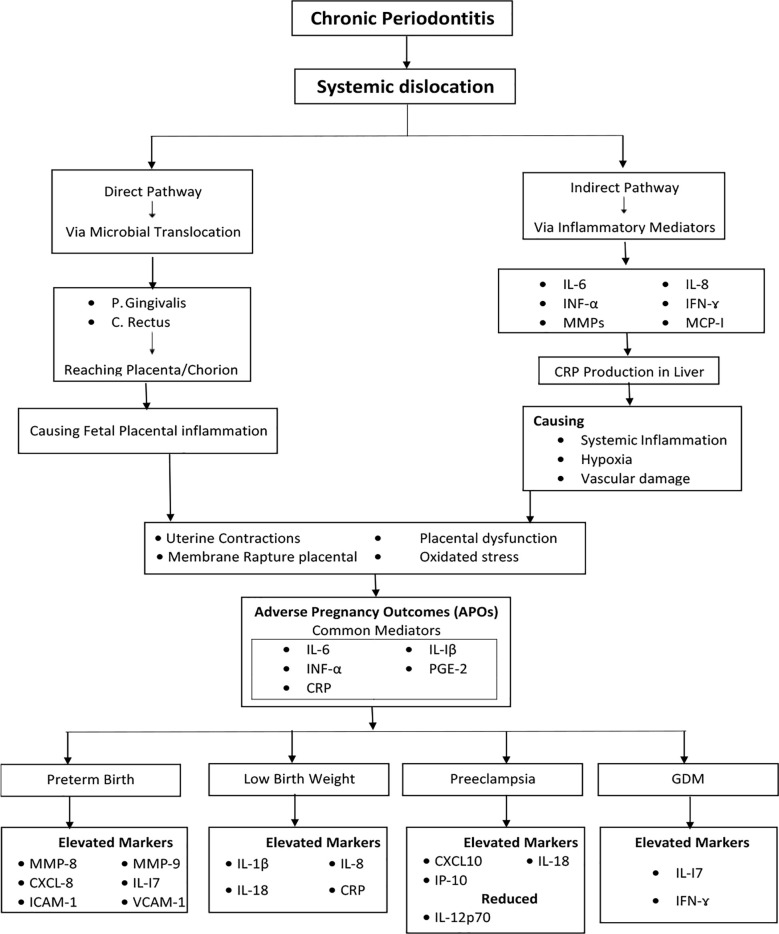
Established association linkages between chronic periodontitis and Adverse Pregnancy Outcomes.

### Data Collection Process & Data Items:

Two reviewers independently gathered data through a standardized form. The form was pilot-tested on a subset of studies. Data were entered in the form using Google Docs and discrepancies were resolved through discussion. The form included the following items:

- ***Names of Inflammatory mediators:*** Specific chemokines, cytokines and other inflammatory molecules measured
- ***Mechanisms:*** Reported biological pathways that link periodontal infection/inflammation to adverse pregnancy outcomes.
- ***Study characteristics:*** Authors, title, journal, year of publication, sample size, study design and population details.
- ***Adverse pregnancy outcomes:*** preterm deliveries, GDM, preeclampsia, stillbirth, LBW and others.

### Study Risk of Bias Assessment:

The quality assessment of each study, which progressed to the “include decision,” was done independently by two reviewers (Ayesha Sadiqa and Zahra Sakina) using various tools. Non-randomized clinical trials were assessed via the ROBINS-I (Risk of Bias in Non-Randomized Studies - of Interventions) tool. The Newcastle-Ottawa Scale (NOS) was used in observational studies to determine the quality of selection, comparability and outcome/exposure assessment. Systematic reviews were assessed using the ROBIS (Risk of Bias in Systematic Reviews) tool. Based on these assessments, studies were categorized as low, moderate or high bias risk.

### Effect Measures:

For dichotomous outcomes (e.g., preterm birth, preeclampsia), odds ratios or relative risks were noted. For continuous outcomes (e.g., cytokine concentrations), mean differences or correlation coefficients were extracted. Effect measures were described qualitatively based on the direction and magnitude of the association.

### Synthesis Methods:

Due to the heterogeneity in study designs and outcomes, findings were summarized using a narrative synthesis approach. Type of mediator, pregnancy outcome, study characteristics and key findings were tabulated. Patterns and consistency of associations across studies were described.

### Reporting Bias Assessment:

To find potential reporting bias by searching grey literature, reviewing trial registries and checking the references of included studies for additional reports. Although not formally quantified, the possibility of selective reporting when interpreting results was considered.

### Certainty Assessment:

The confidence in the evidence was evaluated qualitatively by commenting on its strength and consistency. The predominance of observational studies suggests a low overall certainty.

## RESULTS

The systematic review on chronic periodontitis and APOs supported a strong association. Oral infections can contribute to systemic diseases, including those affecting pregnancy. Here, the most common APOs included GDM, preeclampsia, preterm deliveries and LBW. In this respect, the role of cytokines, namely IL-1β, IL-6, TNF-α and PGE2 has been identified in human and animal models within the fetoplacental unit. This happens in two main ways: direct microbial translocation, where pathogens like *C. rectus* and *P. gingivalis* have been found in the amniotic fluid and chorionic tissues, showing they can get past the placental defenses.

The other way is indirect inflammatory mediation, where systemic immune activation occurs. This involves inflammatory mediators like IFN-γ, IL-6, IL-8, TNF-α, MMPs and MCP-1 stimulating the production of CRP in the liver. This can worsen inflammation and hypoxia in the placenta, cause vascular damage and trigger early uterine contractions. Specific mediators such as TNF-α, IL-1β, IL-6, IL-7, IL-8, PGE2, MMP-8, MMP-9, CRP, CXCL8, MCP-1, CCL2, COX-2, sVCAM-1 and sICAM-1 play a part in linking periodontitis to preterm delivery by promoting oxidative stress, stimulating uterine contractions and recruiting macrophages to the inflamed placenta, which worsens its premature rupture.

**Table-III:** Mechanisms by which mediators mediate the adverse pregnancy outcomes in association with chronic periodontitis.

Interlinked Mediators	Involved underlying Mechanisms	Reference
Matrix metalloproteinase-8 (MMP-8)	High MMP-8 levels have been linked with intra-amniotic inflammation and adverse pregnancy outcomes (APOs).	4
Lipopolysaccharides (LPS) induced IL-6 and IL-8	*P. gingivalis*, the causative agent of chronic periodontitis, produces an exotoxin called lipopolysaccharides (Pg-LPS) linked to APOs. Pg-LPS elevates IL-6 and IL-8 levels in chorion-derived cells by signaling TLR-2 cells.	6
IL-8	Elevated serum IL-8 was seen in pregnant females with normal blood pressure affected by chronic periodontitis during the antepartum and postpartum periods. Reduced IL-8 concentrations were associated with the state of preeclampsia.	7
IL-6, IL-8, IL-1β	Pro-inflammatory mediators like IL-1β and IL-8 initiate labor, which induces uterine contractions.	9
IL-12 p-70	Serum IL-12 p-70 has been downregulated in patients with preeclampsia and periodontitis in the antepartum and puerperium.	12
IL-1α, IL-2, IL-5, IL-7, IL-8, IL-9, IL-12, IL-17, IL-18	High IL-1α, IL-2, IL-5, IL-7, IL-8, IL-9, IL-12 and IL-18 levels lead to systemic inflammation, which consequently leads to APOs.
IL-1β, Prostaglandin (PGE2), Tumor Necrosis Factor-alpha (TNF-α)	Increased IL-1β levels induce preterm labor by upregulating PGE2 in response to periodontal pathogens. TNF-α and PGE2 both promote preterm labor by triggering uterine contractions.
IL-6	Elevated IL-6 levels are linked to preterm birth and pre-eclampsia; it crosses the fetoplacental unit and disrupts fetal development, leading to low birth weight (LBW) deliveries.
IL-4, IL-10, IL-13	Imbalances in IL-4, IL-10 and IL-13 levels can cause immune dysfunction, causing APOs.
Matrix Metalloproteinases (MMPs)	Elevated MMP levels cause damage to periodontal tissues as well as fetal membranes, leading to preterm birth.
IL-1β, IL-6, TNF-α, PGE2	Excess TNF-α, IL-6, PGE2 and IL-1β levels in the FPU lead to premature birth or even spontaneous abortion.	14
	Translocation of *P*.* Gingivalis* to pregnant mice’s placental tissues exhibited elevated TNF-*α,* IL-6, IL-17 and IL-1β.	15
C-reactive protein (CRP)	CRP is raised in APOs such as preterm birth, intrauterine growth restriction (IUGR), preeclampsia and GDM
TNF-*α,* IL-1, IL-6 and IL-8	Elevated maternal serum levels of TNF-α, IL-1, IL-6 and IL-8 have been linked to LBW and premature labor.
IL-6, TNF-α	IL-6 and TNF-α work as insulin antagonists, disrupting insulin signaling and carbohydrate metabolism in GDM.
TNF-α, CRP and IL-6	System inflammation due to periodontitis results in elevated TNF-α, CRP AND IL-6 in women with GDM.	18
IL-1β, PGE2	A strong correlation was noted between periodontal inflammatory mediators, i.e., IL-1β and PGE2 and preterm births.	19
Monocyte Chemoattractant Protein-1 (MCP-1), C-C Motif Chemokine Ligand 2 (CCL2)	Elevated MCP-1 levels promote inflammation and recruit macrophages to the placenta.	20
IL-1β, TNF-α	Gingival cells detect bacterial components, such as LPS, through toll-like receptors and IL-1β. In response, these cells produce TNF-α. These cytokines may cross the placental barrier and trigger preterm and LBW deliveries.	23
PGE, TNF-α, IL-1 and IL-6	In response to elevated PGE, TNF-α, IL-1 and IL-6, periodontitis causes the liver to produce more CRP, especially in early pregnancy.	24
CRP	High CRP contributes to an increased risk for APOs like pre-eclampsia and IUGR.	25
Raised IL-12p70, IL-1β, IL-6 and IL-10 before treatment. Elevated TNF-α, MCP-1 and IL-8 post-treatment	Periodontal scaling and root planning reduced gingival crevicular fluid (GCF) levels of IL-12p70, IL-1β, IL-6 and IL-10 at 28 weeks of gestation compared to the control group. While TNF-α, MCP-1 and IL-8 levels increased after treatment.	26
IL-12	Elevated IL-12 levels have been linked to preterm deliveries.
CXCL8, IL-1β, TNFα and IL-6	Elevated CXCL8, IL-1β, TNFα and IL-6 are linked with preterm deliveries.
IL-4,IL-10	Low levels of IL-4 and IL-10 are seen in women experiencing spontaneous abortions, recurrent miscarriages and infertility.
TNF-α, IL-2, IL-4, IL-6 and IL-10	Periodontal patients at greater risk of preterm deliveries had elevated TNF-α, IL-2, IL-4, IL-6 and IL-10 levels.	29
IL-1β, IL-8	Expectant mothers with compromised periodontium had a greater risk of premature uterine contractions and early onset of labor.	30
CRP	High CRP levels in the first trimester of gestation are linked to preterm delivery.	31
Interferon-gamma (IFN-γ),IL-1β and TNF-α	Low salivary IL-1β and IFN-γ during pregnancy and high TNF-α levels have been strongly associated with gestational periodontitis.	32
PGE2, IL-6	Women experiencing preterm birth had the worst periodontal parameters and elevated PGE2 and IL-6.	33
MMP-9	Elevated MMP-9 levels are linked to Preterm Premature Rupture of Membranes (PPROM).	34
IL-6, CRP and TNF-α	IL-6, CRP and TNF-α in females with preeclampsia were elevated in comparison to the controls.	35
IL-8, TNF α and IL 1β	pPROM releases IL-8, TNF α and IL 1β elevating prostaglandins and matrix-degrading enzymes, stimulating uterine contractility.	36
IL-17	Elevated IL-17 levels lead to oxidative stress and increased placental inflammation and are associated with preeclampsia.	38
TNF-α, IL-8	*P. gingivalis*, the prime causative pathogen of periodontitis, raises serum IL-8 and TNF-α, linking periodontitis and GDM.	44
IL-6, IL-8	Systemic invasion of *P. gingivalis* in chorionic tissues in expectant mothers stimulates IL-6 and IL-8 manufacture through toll-like receptor-2 (TLR-2)	45
IFN-γ, IL-8	*P. gingivalis* promotes secretion of IFN-γ and IL-8 in placentaltrophoblast cells.	46
Toll-like receptor-4 (TLR-4)	*F. nucleatum* promotes inflammation of placenta by activating TLRs, giving rise to APOs.	47
TNF-α, IL-8, IFN-γ, cyclooxygenase-2 (COX-2)	Periodontal pathogens and their by-products released TNF-α, IL-8, IFN-γ, cyclooxygenase-2 (COX-2) in placental tissues *in vitro* models.	48
IL-6, CRP	Preeclampsia, initiated by periodontitis, results in elevated IL-6 and CRP in early pregnancy.	49
TNF-α	A rise in mean arterial pressure expressed a two-fold increase in plasma TNF-α in pregnant rodents, causing TNF-α induced hypertension.	50
C-X-C motif chemokine ligand 10 (CXCL10)	Elevated CXCL10 cause placental inflammation and are associated with preeclampsia.	51
IL-18	Elevated IL-18 levels are linked with preeclampsia and IUGR.	52
soluble intercellular adhesion molecule-1 (sICAM-1)	Raised levels of sICAM-1 were found in pregnant women who had preeclampsia and periodontitis.	53
soluble Vascular Cell Adhesion Molecule-1 (sVCAM-1) and sICAM-1	sVCAM-1 and sICAM-1, both adhesion proteins, can be labeled as clinical biomarkers for preterm birth.	54
VCAM-1, MCP-1 and Interferon-gamma-inducible protein 10 (IP-10)	Elevated circulatory VCAM-1, MCP-1 and IP-10 were found in pregnant women with preeclampsia.	55

Immune-inflammatory agents, namely CRP, TNF-α, CXCL10, IL-6, IL-18, sVCAM-1, sICAM-1, IP-10 and MCP-1, have been found in high concentrations in the circulating blood of pregnant individuals diagnosed with preeclampsia who also have periodontitis. In contrast, IL-12 p70 has been found in lower concentrations in preeclamptic patients than in normotensive patients, both of whom have periodontitis.

Cytokines, including TNF-α, CRP, IL-6, IL-17, IL-8 and IFN-γ, are associated with periodontitis and gestational diabetes mellitus. These reactive markers are released in response to the exotoxins of *P. gingivalis* and subsequently disrupt insulin signaling by antagonizing insulin. Similarly, low birth weight labor is linked to periodontitis through intermediary pro-inflammatory cytokines and chemokines, namely TNF-α, IL-1β, CRP, IL-1, IL-6 and IL-18. These inflammatory agents enter the systemic circulation from the focal periodontal inflammatory site. They are deposited at the fetal membrane, hindering intrauterine fetal growth within the placenta, ultimately leading to smaller size and low-weight births.

## DISCUSSION

### Mediating systemic effect of periodontitis:

Since the 1990s, the bidirectional association between systemic conditions and periodontal health has been gaining significant focus from global researchers, especially after the emergence of periodontal medicine.^12^,^13^ The induction of labor is initiated by a surge in specific inflammatory mediators like TNF-α, IL-1β and PGE-2.^13^ Host’s immune response is activated when LPS binds to TLRs and consequently activates macrophages.^19^ As a result, TNFα, PGE2 and IL-1β are released, which may cross the placental barrier.^19^,^23^ These mediators initiate an inflammatory cascade that induces uterine inflammation, potentially resulting in preterm labor and low birth weight babies.^13^,^23^

### Role of pro-inflammatory mediators in APOs:

Additionally, periodontal inflammation triggers pro-inflammatory cytokine (TNF-α, PGE2, IL-1 and IL-6) release, which enters the systemic circulation and transmit their immune-intensive response.^6^ These mediators also induce the production of CRP (an acute-phase reactant) in the liver.^11^,^24^ Elevated CRP levels have also been linked to APOs, including preterm birth, pre-eclampsia, intrauterine growth restriction (IUGR) and GDM.^10^,^25^ Furthermore, excess IL-6, IL-1β, PGE2 and TNF-α, levels in the fetal-placental unit have been linked to premature birth and spontaneous abortion.^14^,^19^ Inflammatory mediators like IL-1β, IL-6, PGE2 and TNF-α also promote Monocyte Chemoattractant Protein-1 (MCP-1) release. Elevated MCP-1 levels cause excessive recruitment of macrophages to the placenta, promoting uterine inflammation.^20^,^26^

### Association of periodontitis with preterm births:

Gestation is a physiological condition with elevated oxidative stress caused by high metabolic rate in placental cells and enormous superoxide secretions.^20^ Preterm delivery occurs earlier than 37 weeks of gestation and globally impacts roughly 2% of pregnancies.^10^,^27^ Pre-term babies have shown an increased tendency to blindness, cerebral palsy, chronic lung disease, deafness and behavioral disorders.^4^,^16^ Preterm birth has been linked to high levels of circulating TNFα, IL-6, IL-1β and CXCL8.^16^,^26^ These inflammatory mediators, in turn, stimulate prostaglandin production (significantly PGE2) in the placental tissues. High PGE2 levels can stimulate uterine contractions prematurely and induce preterm labor.^13^,^28^

Studies have shown that periodontal patients at a greater risk of preterm birth had elevated TNF-α, IL-2, IL-4, IL-6 and IL-10.^29^ They also exhibited poor periodontal health with significantly higher IL-6 and PGE2, which are both known to trigger labor prematurely and play a part in preterm deliveries.^28^,^30^ In early pregnancy, elevated levels of CRP, a marker for systemic inflammation, are also believed to be linked to a high risk of preterm delivery.^31^ CRP primarily induces the Opsonization of causative bacteria and down-regulation of Polymorphonuclear Neutrophils (PMNs).^5^,^31^ Additionally, reduced IFN-gamma and salivary IL-1β and elevated TNF-α levels are observed during gestation, suggesting that periodontal inflammatory mediators may contribute to the pathophysiology of gestational outcomes.^32^

### Common Mediators of Periodontitis and Labor Inducement:

High levels of IL-6, IL-1β, , PGE2, TNF-α, α-fetoprotein and fibronectin in the amniotic fluid are strongly linked to the onset of labor inducement.^19^ Additionally, high levels of matrix metalloproteinases (MMPs),^13^ such as MMP-2, MMP-8, MMP-9 and MMP-13 have been linked to periodontal destruction as well as preterm birth.^19^,^33^ Raised levels of MMP-9 are strongly linked to Premature Rupture of Membranes (PROM).^34^ IL-6 and TNF-α work as insulin antagonists, disrupting insulin signaling and carbohydrate metabolism in GDM and this condition also one of the leading cause of premature births.^16^ IL-6, CRP and TNF-α in females who have pregnancy-induced hypertension are elevated in comparison to the controls.^16^,^35^ Periodontitis-associated PROM releases IL 1β, IL-8 and TNF-α, which elevate prostaglandins and matrix-degrading enzymes, stimulating uterine contractility.^36^

### Periodontitis and Gestational DM:

Expectant mothers with periodontitis were at a 4.1 times higher risk of experiencing spontaneous abortions.^37^ Elevated IL-17 levels lead to oxidative stress and increased placental inflammation and are associated with preeclampsia.^38^ Expectant mothers with GDM have worse periodontal health and elevated ROS expression than expectant mothers without GDM.^39^ Expectant mothers with periodontitis are twice as likely to develop GDM.^40^ According to Andonova et al. (2015), chronic periodontitis could mediate its systemic effect in three ways: migrating infective periodontal agents to foetal membranes, reactive modulation of LPS on FPU and the response of immune-inflammatory cytokines on placental membranes.^41^

*P. gingivalis* increases reactive oxidative stress (ROS), which induces hypoxia in the PFU, playing a role in inflammation of the uterus and endothelial cell damage.^42^ Enhanced oxidative stress and reduced antioxidants in the last trimester also increased the deterioration of periodontium.^43^ *P. gingivalis*, the primary pathogen responsible for periodontitis, raises serum IL-8 and TNF-α, linking periodontitis to GDM.^44^ *P. gingivalis* in chorionic tissues of high-risk expecting mothers induces IL-6 and IL-8 production through toll-like receptor-2 (TLR-2).^45^ *P. gingivalis* promotes IFN-γ and IL-8 secretion in placental trophoblast cells.^46^ Similarly, *F. nucleatum* induces inflammation of the placenta by activating TLRs, leading to APOs.^47^ Periodontal pathogens and their by-products released TNF-α, COX-2, interferon-γ (IFN-γ) and IL-8 in placental tissues in vitro models.^48^

### Periodontitis association with Preeclampsia:

Preeclampsia, initiated by periodontitis, results in elevated IL-6 and CRP in early pregnancy.^49^ A rise in mean arterial pressure showed that plasma levels of TNF-α increased by two-fold in pregnant rodents, causing TNF-α induced hypertension.^50^ Elevated CXCL10 levels cause placental inflammation and are associated with preeclampsia.^51^ Elevated IL-18 levels are linked with preeclampsia and IUGR.^52^

Pregnant women with poor periodontal parameters often exhibit increased levels of IL-1β and IL-8, resulting in premature uterine contractions that will result in premature labor.^17^,^45^ Raised sICAM-1 levels were found in those pregnant women who had periodontitis as well as preeclampsia.^53^ Another similar study claimed that sICAM-1 and sVCAM-1 could be considered clinical biomarkers for preterm birth.^54^ Molvarec et al., in the year 2013 on the same lines, reported the same findings along with the raised levels of IP-10, VCAM-1 and MCP-1 in preeclamptics compared to normotensives.^55^

### Suggestive approaches to reduce the risk of APOs:

Literature also recommends that pre-conception time is the optimal period for periodontal treatment for better gestational outcomes and for the downregulation of the risk of APOs.^26^,^56^ A study noticed that periodontal treatment lowered the gingival crevicular fluid (GCF) levels of IL-6, IL-10, IL-12p70 and IL-1β mediators at the 28^th^ week of gestation in comparison to the control group while TNF-α, IL-8 and MCP-1 levels were elevated after treatment. Periodontal treatment and root scaling in expectant mothers also lower the risk of preterm deliveries and LBW.^26^,^57^

## CONCLUSION

Chronic periodontitis is significantly associated with APOs, including preterm deliveries, GDM, preeclampsia and LBW. Periodontal pathogens and by-products translocate to the fetoplacental unit or trigger systemic inflammation through immune mediators like PGE2, IL-1β, IL-6, IL-8, TNF-α and CRP. These agents cause uterine inflammation, placental hypoxia and disrupted insulin signaling linked to complications. Specific periodontal pathogens activate TLRs, intensifying placental inflammation that upregulates sICAM-1, sVCAM-1, MCP-1, IL-17, MMPs and CXCL10 implicated in preeclampsia. TNF-α and IL-6 also disrupt insulin signaling in GDM. The review advocates early diagnosis and management of periodontal disease, especially during preconception, to reduce APO risks. Periodontal scaling and root planing reduce inflammatory markers and improve pregnancy outcomes. Promoting oral health as part of prenatal care will protect maternal and fetal well-being.
